# Clinical feasibility of accelerated whole liver water T_1_ mapping with T_2_*-compensation

**DOI:** 10.1186/s41747-026-00689-z

**Published:** 2026-03-04

**Authors:** Elizabeth Huaroc Moquillaza, Lisa Steinhelfer, Kilian Weiss, Robert Walter, Jonathan Stelter, Mariya Doneva, Rickmer Braren, Dimitrios C. Karampinos

**Affiliations:** 1 Institute for Diagnostic and Interventional Radiology, School of Medicine and Health, TUM University Hospital, Technical University of Munich, Munich, Germany; 2 Philips GmbH, Market DACH, Hamburg, Germany; 3 Philips Innovative Technologies, Hamburg, Germany; 4 German Cancer Consortium, a Partnership Between DKFZ and School of Medicine, Technical University of Munich, Munich, Germany; 5 Laboratory of Magnetic Resonance Imaging Systems and Methods, Ecole Polytechnique Fédérale de Lausanne (EPFL), Lausanne, Switzerland; 6 CIBM Center for Biomedical Imaging (CIBM), Lausanne, Switzerland

**Keywords:** Biomarkers, Carcinoma (hepatocellular), Liver fibrosis, Liver neoplasms, Magnetic resonance imaging

## Abstract

**Objective:**

Current liver T1 mapping methods present restricted liver coverage, take long acquisition times and mostly exclude the T1 bias induced by fat and iron effects. We evaluated the clinical feasibility of an accelerated water T1 (wT1) mapping method, including all liver segments and the potential of its T2*-compensation (wT1_comp_) for fibrosis tissue assessment.

**Materials and methods:**

Forty-three patients were classified into three groups: benign without/with risk of developing fibrosis and hepatocellular carcinoma (HCC). A 9-slice accelerated single-shot spiral continuous inversion-recovery Look-Locker (CIR-LL) wT1 mapping acquisition, performed in an 11-s breath-hold, and clinical images (proton density fat fraction (PDFF), T2*, T1- and T2-weighted) were acquired for all patients. ROIs were defined on the PDFF, T2* and wT1 maps in all liver segments. wT1_comp_ was estimated based on the wT1-T2* correlation of the benign-no-risk group and was compared to wT1 and clinical images inspecting for fibrosis.

**Results:**

For each patient group, wT1 maps presented broad liver coverage, capturing all liver segments. T2* and wT1 measurements of the benign-no-risk group were significantly correlated $({{{\rm{wT}}}}1=12.78* {{{{\rm{T}}}}2}^{* }+481.45{{{\rm{;\; r}}}}=0.78,\,p \, < \,0.001)$ and the T2*-compensation model was defined by ${{{{\rm{wT}}}}1}_{{{{\rm{comp}}}}}={{{\rm{wT}}}}1{minus}\,12.78* ({{{{\rm{T}}}}2}^{* }\,{minus}\,22)$. Liver segments of the same patient presented different wT1 values. Outperforming wT1, wT1_comp_ identified 21 liver segments from nine patients associated with qualitative fibrosis findings in clinical images, some only visible in post-contrast T1-weighted images.

**Conclusion:**

The wT1 method is feasible for fast broad liver coverage in patients with HCC or benign lesions. The segments-based wT1_comp_ analysis shows potential for noninvasive contrast-free qualitative liver fibrosis assessment.

**Relevance statement:**

The proposed water-specific T1 mapping method, its T2*-compensation and the inclusion of all liver segments could be clinically relevant for the tissue signal assessment of fibrotic liver segments without contrast agent administration.

**Key Points:**

- The developed water T1 (wT1) method enables broad liver coverage in a single 11-s breath-hold.
- Liver wT1 mapping and the proposed T2*-compensation (wT1_comp_) remove the bias in T1 induced by fat and iron, respectively.
- The analysis in all liver segments allows the assessment of focal liver changes.
- The proposed liver segments-based wT1_comp_ method presents potential to identify tissue signal changes associated with fibrosis.

**Graphical Abstract:**

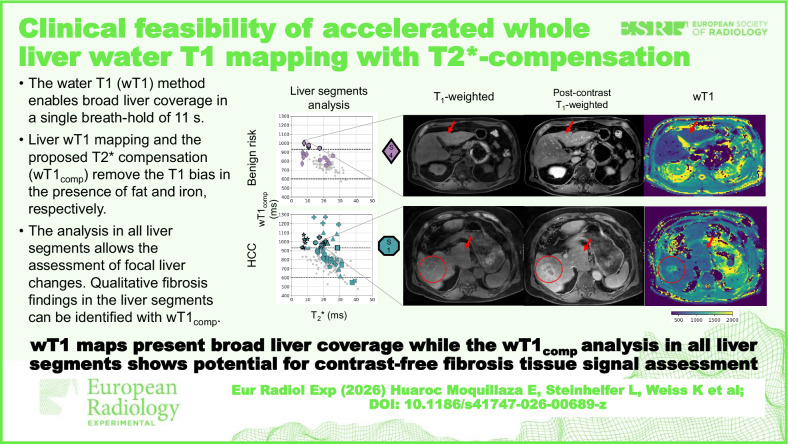

## Background

T1 mapping has emerged as a promising magnetic resonance imaging (MRI) biomarker for the assessment of liver diseases, mainly for fibrosis, cirrhosis, and focal lesions. It presents satisfactory diagnostic performance in identifying liver fibrosis in comparison to biopsy [1]. Studies have demonstrated that T1 is significantly longer for patients with fibrosis [2, 3] and cirrhosis [4, 5] in comparison to a healthy cohort.

T1 relaxation time prolongs with liver fibrosis due to structural and pathophysiological changes. Excess extracellular matrix deposition, edema, and inflammation increase extracellular water content, modifying spin-lattice interactions and prolonging T1. Inflammation further contributes to vascular permeability and tissue swelling, altering water mobility. As fibrosis advances, these effects intensify, leading to a progressively prolonged T1 relaxation time [6, 7]. The potential of T1 for the staging of liver fibrosis has also been evaluated in combination with Fibroscan [8] or magnetic resonance elastography (MRE) [9], which is the most accepted MRI-based method for fibrosis evaluation [10, 11].

Moreover, T1 mapping has been applied for the differentiation of benign and malignant liver lesions based on the change of the T1 value after contrast agent uptake [12–15]. T1 mapping is valuable for distinguishing focal liver lesions based on their unique histological properties. Between focal liver lesions, benign lesions, such as cysts and hemangiomas, typically exhibit longer T1 relaxation times than malignant lesions like hepatocellular carcinoma (HCC) and metastases [13]. The use of contrast agents further enhances diagnostic accuracy by enabling quantitative analysis of pre- and post-contrast T1 values. These measurements provide important insights into lesion composition and aid in differential diagnosis.

Most of the T1 mapping methods used in the aforementioned studies propose a restricted liver coverage and still require long acquisition times, compromising its further clinical applicability. Previous 2D T1 mapping studies for fibrosis and cirrhosis acquired between one [3] to four [9] slices in 12–14 s and 4.5 s per slice, respectively. Despite the restricted number of slices, the liver was characterized using ROI analysis: a single ROI in S8 [1, 3], multiple ROIs in a single slice targeting the liver lobes [1, 5] or the visible liver segments [2] and the whole liver segmentation in a single slice [1, 9] or few slices [1]. Previous two-dimensional (2D) T1 mapping methods used for the liver lesions studies acquired one [14] to five [13] slices in 20‒30 s and 2.7 s per slice, respectively, and used a region of interest (ROI) in the lesion for the analysis. Therefore, current 2D T1 mapping methods lack an extensive liver coverage.

A broad liver coverage would enable a more comprehensive assessment of each hepatic segment, improving the detection of focal parenchymal changes. Capturing a large tissue volume allows for a more accurate characterization of liver lesions, facilitating the differentiation between benign and malignant findings. Additionally, a broad liver coverage provides valuable insights into disease progression and treatment response, ensuring a more detailed and reliable evaluation of hepatic pathology. As an alternative, the three-dimensional (3D) variable flip angle T1 mapping methods offer whole liver coverage [4, 8, 12, 15]. However, they are prone to ${{{{\rm{B}}}}}_{1}^{+}$ inhomogeneities and require long breath-holds of 18–20 s, overstraining the patients.

T1 is known to be biased by iron and fat. In the presence of iron in the liver, the T2* effect in T1 increases and can be removed using an additional T2* map and Bloch simulations [3, 16]. Water T1 (wT1) mapping methods minimize the influence of fat on liver T1 [17], estimating it accurately in patients [17–21]. However, wT1 methods evaluated in clinical cohorts still present restricted liver coverage, long scan duration and are sensitive to ${{{{\rm{B}}}}}_{1}^{+}$ inhomogeneities. These restrictions remain to a lesser extent in new liver wT1 mapping technical developments [22–30], which also lack clinical feasibility evaluations in patients with different diagnoses. In particular, despite the whole liver coverage, the recent 3D wT1 mapping methods [17, 23–28] still require long acquisition times (from 0:17 to 6:09 min:s) or are fast, but need ${{{{\rm{B}}}}}_{1}^{+}$ compensation [29, 30]. In contrast, from the new 2D methods [22, 31], the work in [31] allows the fastest ${{{{\rm{B}}}}}_{1}^{+}$-robust liver wT1 mapping of 9 slices in a short-breath-hold of 11 s. The compensation of both iron and fat biases has already been addressed [32–34]; however, previous works were based on the acquisition of one [32, 34] and three [33] slices, facing the lack of liver segments coverage described above.

Based on the previous evidence, an accelerated method for T2*-compensated wT1 (wT1_comp_) mapping with broad liver coverage would be of advantage. This work proposes to evaluate the clinical feasibility of the spiral-based continuous inversion recovery Look-Locker (CIR-LL) wT1 mapping method [31] and the potential for fibrosis tissue signal assessment of its compensation for T2* bias, analyzing all liver segments.

## Materials and methods

### Patients

Forty-three patients, aged 67.3 ± 11.7 years (mean ± standard deviation (SD)), range 35‒86 years, 26 males and 17 females, who were scheduled for an abdominal MRI scan from August 2023 to May 2024, were prospectively enrolled for this study. No specific exclusion criteria were applied for patient selection. The cohort consisted of 3 groups: 9 patients with benign lesions (benign-no-risk-group), including 5 intraductal papillary mucinous neoplasms (IPMN), 2 hemangiomas and 2 no structural alterations; 11 patients with benign lesions under risk of developing fibrosis (benign-risk group), including 6 IPMNs, 1 hemangioma, and 4 no structural alterations; and 23 patients with HCC diagnosis (HCC group) per documentation in the local hospital information system. Following Thompson et al [33], a patient with benign lesions was included in the benign-risk group if the patient presented dyslipidemia, type 2 diabetes, body mass index > 30 kg/m^2^, or mean proton density fat fraction (PDFF) of all liver segments > 5%. Conversely, if a patient with benign lesions presented no dyslipidemia, no type 2 diabetes, body mass index ≤ 30 kg/m^2^ and mean PDFF of all liver segments ≤ 5%, the patient was considered part of the benign-no-risk group. Ethical approval from the local ethics committee was obtained prior to conducting the study, and all participants provided informed consent.

### wT1 mapping methodology

**Fig. 1:**
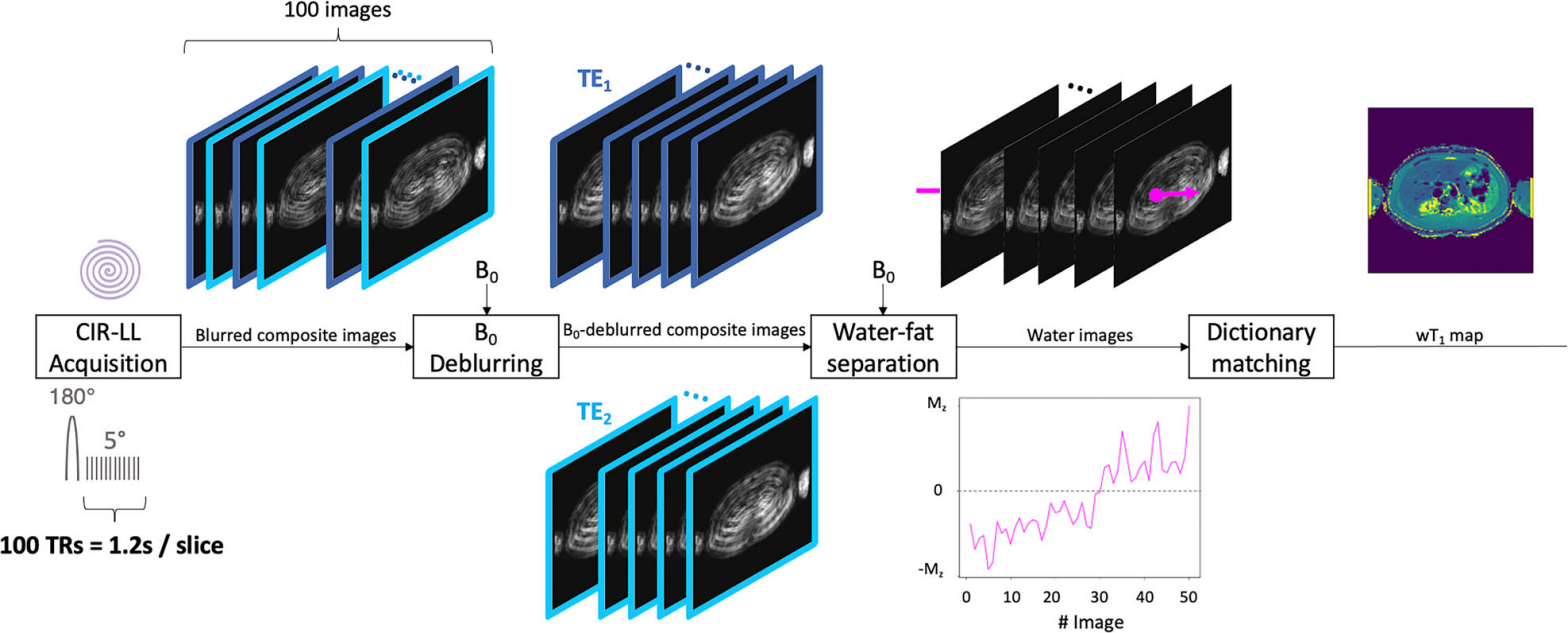
wT1 mapping methodology. Workflow to obtain the wT1 map of a slice. The CIR-LL acquisition is performed in 1.2 s, obtaining 100 undersampled spiral images (50 images × 2 TEs). After B_0_-deblurring and water-fat separation, each 50-length water signal (fuchsia) is matched to a previously computed dictionary to estimate the value of the corresponding pixel in the wT1 map. CIR-LL, Continuous inversion recovery Look-Locker; TE, Echo time; TR, Repetition time; wT1, Water T1

In Fig. 1, we show the wT1 mapping methodology for a single slice proposed in [31]. A continuous inversion recovery Look-Locker (CIR-LL) acquisition has been combined with B_0_-deblurring and water-fat separation to obtain water images, which are then matched to a dictionary to estimate a wT1 map. During the CIR-LL acquisition, 100 composite (water + fat) images were acquired in 1.2 s for the same slice using an adiabatic slice-selective inversion pulse followed by a 5° flip angle train with repetition time of 12 ms. A composite image is acquired per repetition time using a single spiral readout at interleaved echo time (TE): TE_1_/TE_2_ = 2.3/3.3 ms.

The acquired images were blurred because of the use of spiral trajectories in the presence of off-resonance and show strong undersampling artifacts. To correct for the B_0_-blurring, a B_0_ map is acquired as a pre-scan and is used to perform B_0_-deblurring on the 100 composite images, which are grouped per TE into two groups of 50 images. As a next step, water-fat separation [35] was performed on every TE image pair using the B_0_ pre-scan and a 7-peak [36]. Finally, the resulting 50 water images went through the dictionary matching step to estimate the wT1 map. Using the Extended Phase Graph method [37–39], the dictionary contains a collection of simulated signal evolutions after the application of the proposed pulse sequence for the range T1 = [100, 3,000] ms in steps of 5 ms, T2 = 50 ms and ${{{{\rm{B}}}}}_{1}^{+}$ = 1. T2 and ${{{{\rm{B}}}}}_{1}^{+}$ were set to a specific value since the wT1 mapping method does not encode T2 and is robust to ${{{{\rm{B}}}}}_{1}^{+}$ inhomogeneities. The matching is performed by computing the complex inner product between every 50-length signal of a water pixel and all dictionary signals, both normalized. The T1 of the dictionary vector that corresponds to the highest inner-product value is assigned as the wT1 at the corresponding pixel.

For multislice wT1 mapping, the methodology shown in Fig. 1 is repeated per slice in an interleaved slice order.

### MRI protocol

All measurements were performed on a 3-T Ingenia Elition X scanner (Philips Healthcare) with the sequence parameters summarized in Table 1. As part of the clinical routine, T2-weighted and T1-weighted images (before and after contrast injection), as well as a chemical shift encoding-based water-fat separation method using the vendor’s mDixon quant product to measure PDFF and T2* maps, were acquired for the whole liver. As part of the proposed wT1 method, low-resolution B_0_ maps were acquired in free-breathing and were interpolated to the acquired image resolution. Additionally, the proposed CIR-LL acquisition was performed 10 s after the B_0_ pre-scan in an 11-s breath-hold covering nine liver slices scanned in an interleaved order (slice 1, 4, 7, 2, 5, 8, 3, 6, 9). For each slice, the acquired 100 images were processed to estimate the wT1 maps according to the workflow in Fig. 1.

**Table 1:** Sequence parameters

				Proposed wT1 method acquisition	
	T2-weighted	T1-weighted	PDFF T2*	B_0_ pre-scan	CIR-LL
**Trajectory**	MultiVane	Cartesian	Cartesian	Cartesian	Spiral (Uniform sampling density)
**Spiral readout** ${{\boldsymbol{T}}}_{{\boldsymbol{acq}}}$ **(ms)**	-	-	-	-	5.6
**Scan mode**	Multislice	3D	3D	3D	2D
**Technique**	Spin-echo	Spoiled gradient-echo	Dixon spoiled gradient-echo	Dixon Gradient-echo	Spoiled gradient-echo
**Field of view** **(mm**^**2**^**)**	400 × 400	400 × 400	400 × 400	500 × 500	450 × 450
**Acquisition voxel size (mm**^**2**^**)**	1.25 × 1.25	1.47 × 1.47	2.50 × 2.50	3.13 × 3.13	2.81 × 2.81
**Slice thickness (mm)**	4	5	6	3	10
**Slices**	60	106	25	50	9
**Gap (mm)**	0.4	-2.5	-3	0	1
**TE (ms)**	127	1.31, 2.4	0.97, 1.67, 2.37, 3.07, 3.77, 4.47	1.32, 2.46, 3.60, 4.74	TE_1_ = 2.3 TE_2_ = 3.3
**TR (ms)**	2080	3.7	5.6	6	12
**Flip angle** ${{\boldsymbol{\alpha }}}$ **(°)**	90	10	3	10	5
**Respiratory** **compensation**	Trigger	Breath-hold	Breath-hold	-	Breath-hold
**Flow compensation**	No	No	No	No	Yes
**Acceleration**	SENSE R = 2.5	CS-SENSE R = 6	CS-SENSE R = 4	SENSE R = 2	Undersamp. R = 15
**Total scan time (s)**	~240 (2.1 per respiratory cycle)	12.6 per breath-hold	9	12.8	11

*2D* Two-dimensional, *3D* Three-dimensional, *CIR-LL* Continuous inversion recovery Look-Locker, *CS* Compressed sensing, *PDFF* Proton density fat fraction, *SENSE* Sensitivity encoding, *TE* Echo time, *TR* Repetition time

### ROI definition

Two radiologists, with 3 and 6 years of experience, independently assessed the quality of the wT1 maps on a PACS workstation (Sectra AB, Linköping, Sweden), analyzing 22 cases and the whole cohort, respectively. For each patient, they placed a 15-mm circular ROI in each available liver segment (S1, S2, S3, S4a, S4b, S5, S6, S7, and S8) on the PDFF, T2*, and wT1 maps to ensure comprehensive analysis. ROIs were placed independently on all maps, using the standard vendor-specific ROI tools of the PACS system and T2-weighted images as a reference. For the HCC patients, the ROIs were placed in the liver parenchyma, neglecting liver segments if they were occupied by lesions.

### T2*-compensation for wT1

Recently, the work in [33] has shown that liver T2* and wT1 are strongly correlated and has proposed the use of the correlation results in a healthy cohort for compensating for the T2* effect on wT1. Following this, a calibration approach to estimate wT1_comp_ is employed based only on the results of the benign-no-risk group. First, the correlation between T2* and wT1, estimated from the CIR-LL acquisition, is defined for the benign-no-risk group. Then, the slope of the correlation (${{{\rm{m}}}}$) and the T2* mean (${{{{\rm{mean}}}}}_{{{{{\rm{T}}}}}_{2}^{* }}$) value of the benign-no-risk group are used to define the linear compensation model ${{{{\rm{wT}}}}1}_{{{{\rm{comp}}}}}={{{\rm{wT}}}}1\,{minus}{{{\rm{m}}}}({{{{\rm{T}}}}2}^{* }\,{minus}\,{{{{\rm{mean}}}}}_{{{{{\rm{T}}}}2}^{* }})$, where wT1 and T2* refer to measurements of the same anatomical regions on the same subject.

### Statistical analysis

Power calculations using Cohen’s effect size *d* were performed, focused on the cohort size, assuming one measurement per subject, a split of 9 subjects as in the benign-no-risk group *versus* 34 subjects as in the benign-risk and HCC groups, effect size *d* = 0.65 and statistical significance α = 0.05 (two-sided). To evaluate the inter-observer agreement, the intraclass correlation coefficient (ICC) was calculated for T2*, PDFF and wT1 using the 22 cases analyzed by both radiologists. The linear regression and Pearson coefficient $\left({{{\rm{r}}}}\right)$ were used to characterize the linear relationship between the T2* and wT1 measurements of the benign-no-risk group. A test of the two-sided null hypothesis that these T2* and wT1 measurements are uncorrelated and normally distributed was performed, obtaining the corresponding *p*-value. All these calculations were performed in Python using *TTestIndPower* from *statsmodels.stats.power* for the power, *intraclass_corr* with a two-way mixed with fixed raters model from *Pingouin* for the ICC, *LinearRegression* from *sklearn.linear_model* for the linear regression and *pearson.r* from *scipy.stats* for the Pearson coefficient and *p*-value.

The mean, SD and range of the ROIs placed in the PDFF, T2*, wT1 maps and the wT1_comp_ estimations were calculated for each patient group. Using the mean and SD of the benign-no-risk group, a wT1 and wT1_comp_ ROI value was defined as high if ${{{\rm{wT}}}}1 > {{{{\rm{mean}}}}}_{{{{\rm{wT}}}}1}+1.96{{{{\rm{SD}}}}}_{{{{\rm{wT}}}}1}$ and ${{{{\rm{wT}}}}1}_{{{{\rm{comp}}}}} > {{{{\rm{mean}}}}}_{{{{{\rm{wT}}}}1}_{{{{\rm{comp}}}}}}+1.96{{{{\rm{SD}}}}}_{{{{{\rm{wT}}}}1}_{{{{\rm{comp}}}}}}$, respectively, being considered low, otherwise.

### Fibrosis inspection

Fibrosis is strongly associated with hepatic fat accumulation and the development of HCC. Moreover, fibrosis is a key factor contributing to increase in the liver T1 relaxation time [2, 3]. Based on this, liver segments corresponding to ROIs with low wT1, but high wT1_comp_ were inspected in T2-weighted and T1-weighted (before and after contrast agent, Gadobutrol, 0.1 mmol/kg body weight, Gadovist, Bayer Healthcare, Berlin, Germany) images and in wT1 maps by the radiologist with more experience. The liver segments were evaluated for tissue signal changes and reticulations as a qualitative assessment of fibrosis. The analysis focused on these liver segments since, without the T2*-compensation, they would not have been associated with qualitative fibrosis findings given their low wT1 value.

## Results

### Size and wT1 maps of the cohort

**Fig. 2:**
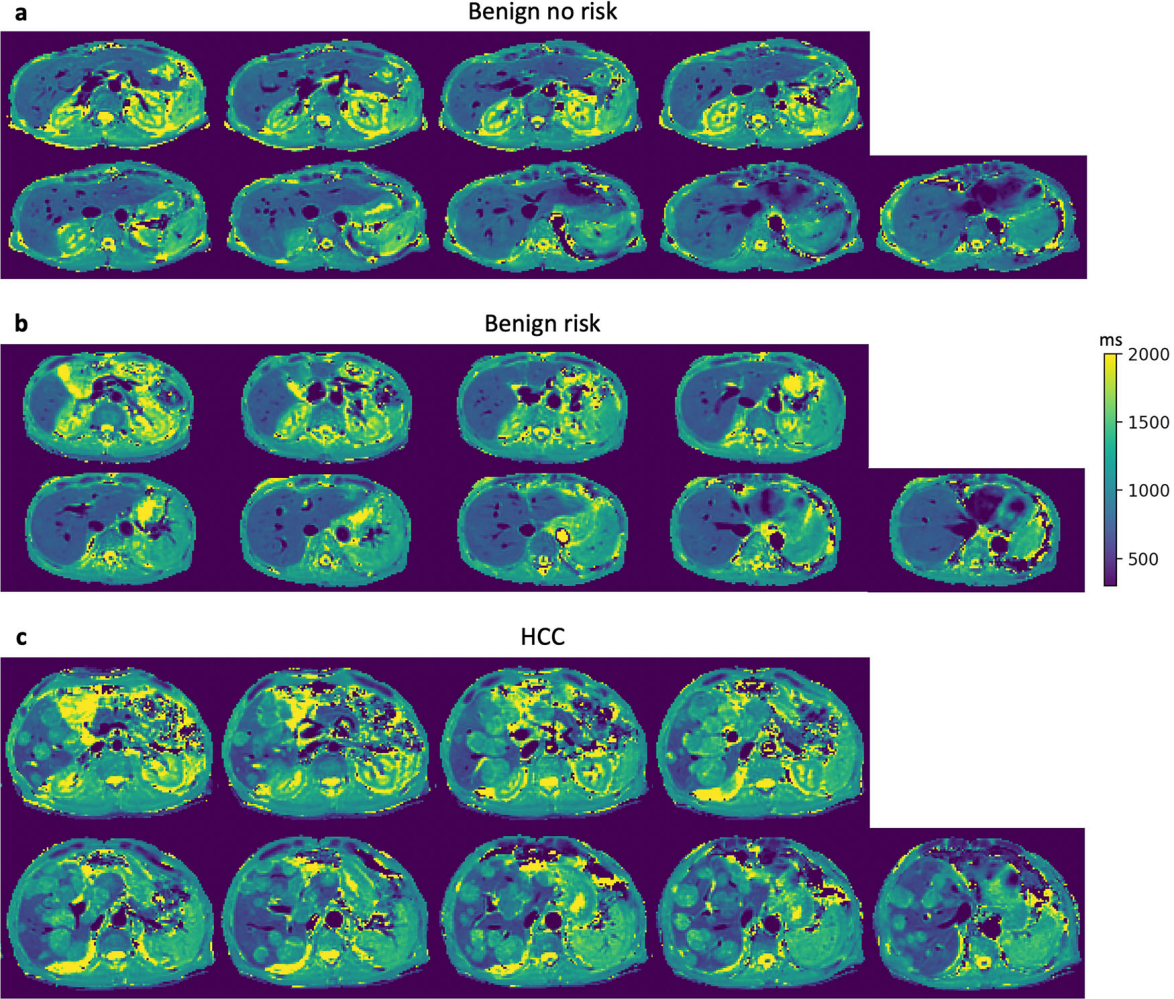
wT1 maps for the three groups in this study. 9-slice wT1 maps acquired in an 11 s breath-hold are shown for one patient in each group. **a** A 45-year-old woman with no pathology. **b** A 75-year-old woman with IPMN diagnostic, with no liver involvement and no signs of malignancy. **c** 74-year-old man with multifocal HCC depicted in great detail by the wT1 maps. HCC, Hepatocellular carcinoma; IPMN, Intraductal papillary mucinous neoplasm; wT1, Water T1

Assuming a reference group of the size of the benign-no-risk group, the estimated power = 40% shows that the cohort size is underpowered. Figure 2 exemplifies the good quality and broad liver coverage of the nine wT1 maps for each patient group. Remarkably, wT1 maps depict HCC lesions with great detail, as shown in Fig. 2c. The inspection of the 387 wT1 maps (43 patients × 9 slices) detected that 13 slices (3%) were affected by motion artifacts. These slices were discarded for the rest of the analysis.

### ROI placement

**Fig. 3:**
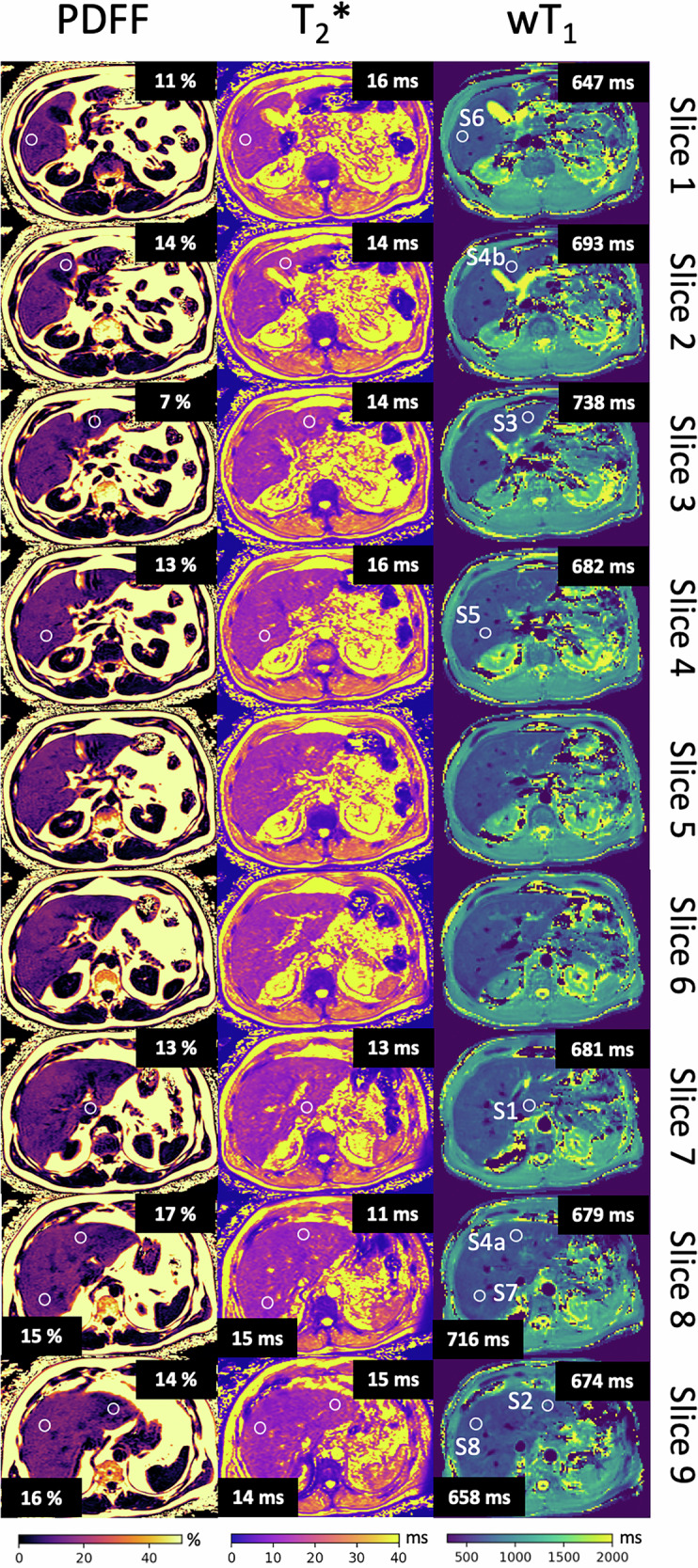
ROI placement. The PDFF, T2* and wT1 maps of a 65-year-old patient of the benign-risk group with no evidence of malignancy are displayed. An ROI (white circle) was placed in every liver segment in the PDFF, T2* and wT1 maps. The nine wT1 maps are shown together with their anatomically corresponding PDFF and T2* maps. For this case, slice 5 and 6 represent the transition between liver segments; thus, no ROI was placed. Thanks to the availability of more slices, all liver segments could be located. The mean value of each ROI is displayed in the dark boxes. The mean value of the PDFF ROIs is higher than 5%; therefore, this case is considered part of the benign-risk group. PDFF, Proton density fat fraction; ROI, Region of interest; wT1, Water T1

Figure 3 shows an example of the ROIs placement and highlights the importance of the multislice characteristic of the wT1 maps to capture all liver segments.

### Model for T2*-compensation for wT1

**Fig. 4:**
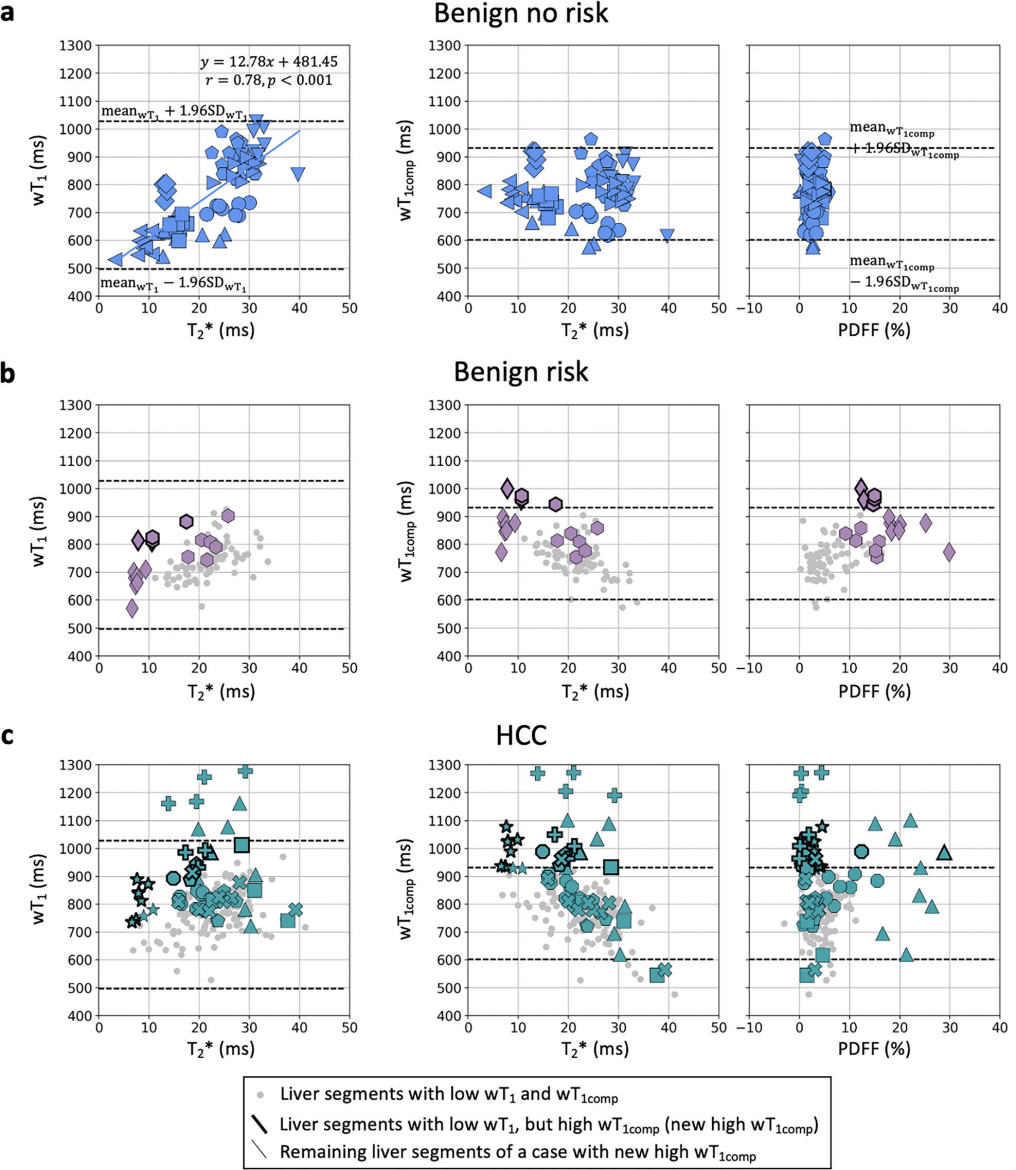
ROI analysis. wT1 *versus* T2* (first column), wT1_comp_ *versus* T2* (second column) and wT1_comp_ *versus* PDFF (third column) measurements are displayed for all groups. Liver segments of the same patient are displayed with the same marker. Based on the ${{{{\rm{mean}}}}}_{{{{\rm{wT}}}}1}$, ${{{{\rm{SD}}}}}_{{{{\rm{wT}}}}1}$, ${{{{\rm{mean}}}}}_{{{{{\rm{wT}}}}1}_{{{{\rm{comp}}}}}}$ and ${{{{\rm{SD}}}}}_{{{{{\rm{wT}}}}1}_{{{{\rm{comp}}}}}}$ of the benign-no-risk group (Table 2), upper and lower limits are defined (dashed lines). **a** Benign-no-risk group. **b** Benign-risk and (**c**) HCC patients with liver segments with low wT1 ($\,{ < {{{\rm{mean}}}}}_{{{{\rm{wT}}}}1}+1.96{{{{\rm{SD}}}}}_{{{{\rm{wT}}}}1}$), but high wT1_comp_ (${ > {{{\rm{mean}}}}}_{{{{{\rm{wT}}}}1}_{{{{\rm{comp}}}}}}+1.96{{{{\rm{SD}}}}}_{{{{{\rm{wT}}}}1}_{{{{\rm{comp}}}}}}$) are highlighted (thick-edged marker). The remaining liver segments of these patients are shown with the same marker (thin-edged marker). HCC, Hepatocellular carcinoma; PDFF, proton density fat fraction; SD, Standard deviation; wT1, Water T1; wT1comp, T2*-compensated wT1

T2* and wT1 measurements of the benign-no-risk group were significantly correlated $({{{\rm{wT}}}}1=12.78* {{{{\rm{T}}}}2}^{* }+481.45{{{\rm{;\; r}}}}=0.78,\,p < 0.001)$. The estimated ${{{\rm{m}}}}=12.78\,{{{\rm{ms}}}}$ and ${{{{\rm{mean}}}}}_{{{{{\rm{T}}}}2}^{* }}=22\,{{{\rm{ms}}}}$ of the benign-no-risk group are similar to the 12.50 ms and 20.00 ms estimated as the correlation slope and mean T2* value from a healthy cohort before T2*-compensation in [33], respectively. Then, the proposed model for T2*-compensation is defined by ${{{{\rm{wT}}}}}_{1{{{\rm{comp}}}}}={{{{\rm{wT}}}}}_{1}{minus}\,12.78* ({{{{\rm{T}}}}}_{2}^{* }{{{\rm{minus}}}}22)$. Figure 4 shows the estimated wT1 w.r.t. T2* together with the estimated wT1_comp_ w.r.t T2* and PDFF for all cases.

### ROI analysis

High inter-observer agreement was found for all parameters (Fig. S1): ${{{{\rm{ICC}}}}}_{{{{{\rm{T}}}}2}^{* }}=0.98\,(95 \% {{{\rm{CI}}}}\,0.98{-}0.99)$, ${{{{\rm{ICC}}}}}_{{{{\rm{PDFF}}}}}=0.99\,(95 \% {{{\rm{CI}}}}\,0.99{-}0.99)$ and ${{{{\rm{ICC}}}}}_{{{{\rm{wT}}}}1}=0.92(95 \% {{{\rm{CI}}}}\,0.90{-}0.94)$. Table 2 shows the statistics of all measurements presented in Fig. 4. Table 2 and Fig. 4 confirm the low PDFF values (2.9 ± 1.3%) for the benign-no-risk group, show the wide PDFF range for the benign-risk ([0.2, 29.8]%) and HCC ([0, 28.9]%) groups and the wide T2* range for all groups (benign-no-risk = [3.1, 39.7] ms, benign-risk = [6.6, 33.6] ms and HCC = [6.6, 54.3] ms). Furthermore, the HCC group presents the highest wT1 (812 ± 111 ms, [528, 1,278] ms) and wT1_comp_ (804 ± 128 ms, [476, 1,273 ms]) values.

**Table 2:** Mean, SD, and range of the ROIs defined in the PDFF, T2*, wT1 and wT1_comp_ maps per patient group

		Patient group		
		Benign-no-risk	Benign-risk	HCC
**PDFF (%)**	Mean	2.9	7.8	4.8
	SD	1.3	5.9	4.9
	Range	[0.1, 5.8]	[0.2, 29.8]	[0, 28.9]
T2* (ms)	Mean	22.3	20.1	22.9
	SD	8.2	6.3	7.1
	Range	[3.1, 39.7]	[6.6, 33.6]	[6.6, 54.3]
**wT1 (ms)**	Mean $({{{{\mathrm{mean}}}}}_{{{{\mathrm{wT}}}}{{1}}})$	762	745	812
	SD (${{{{\mathrm{SD}}}}}_{{{{\mathrm{wT}}}}{{1}}}$)	135	68	111
	Range	[531, 1,026]	[571, 925]	[528, 1,278]
**wT1**_**comp**_ **(ms)**	Mean $({{{{\mathrm{mean}}}}}_{{{{{\mathrm{wT}}}}{{1}}}_{{{{\mathrm{comp}}}}}})$	767	772	804
	SD (${{{{\mathrm{SD}}}}}_{{{{{\mathrm{wT}}}}{{1}}}_{{{{\mathrm{comp}}}}}}$)	84	82	128
	Range	[576, 963]	[575, 1,000]	[476, 1,273]

*HCC* Hepatocellular carcinoma, *PDFF* Proton density fat fraction, *SD* Standard deviation, *wT1* Water T1, *wT1*_*comp*_ T2*-compensated wT1

In Fig. 4, the distribution of the different markers shows how spread the wT1 and wT1_comp_ values of the liver segments in a patient can be. This reinforces the need for an analysis at the liver segment level for a better characterization of the whole liver. Figure 4b shows that five liver segments, corresponding to two benign-risk patients (rhombus and hexagon), presented low wT1, but high wT1_comp_ (thick-edged). The majority of the liver segments of these two patients present low wT1_comp_. If the analysis had been done only with a few ROIs or slices, excluding these high wT1_comp_ liver segments, the liver wT1_comp_ might have been wrongly characterized as generally low. Figure 4c presents 16 liver segments with low wT1 and high wT1_comp_ (thick-edged), corresponding to seven HCC patients (octagon, x, square, pentagon, triangle, cross, and star). In three of these HCC patients (octagon, x, and square), a single liver segment was identified with low wT1 and high wT1_comp_ (thick-edged). The other liver segments presented low wT1_comp_, which might have masked the single one with high wT1_comp_ if not included in the analysis. All high wT1_comp_ liver segments were identified thanks to the T2*-compensation and to the regional analysis at the liver segment level.

### wT1_comp_ for fibrosis inspection

**Fig. 5:**
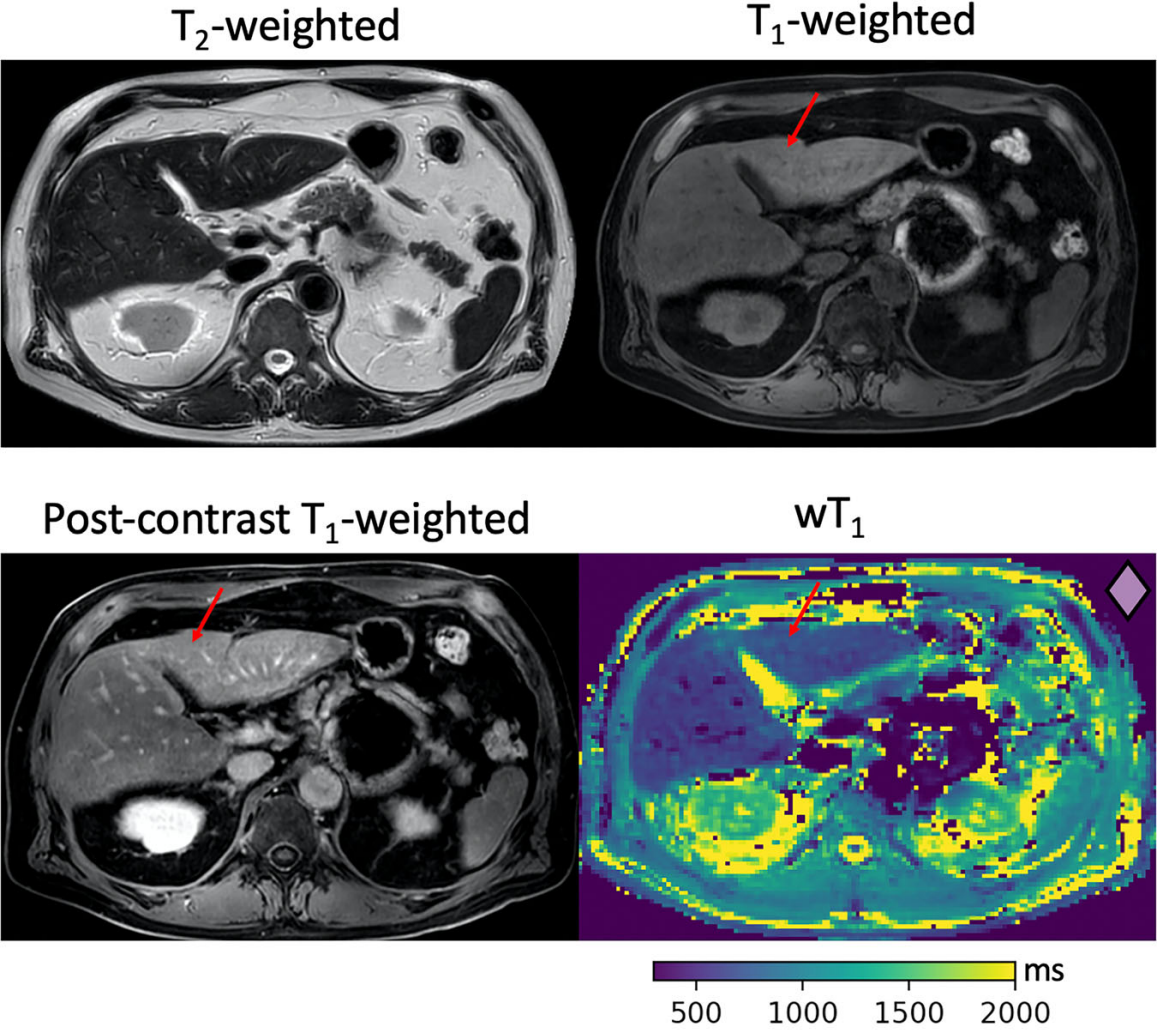
A 62-year-old male with idiopathic granulomatous hepatitis. The T2-weighted image shows no abnormalities. Slight and mild T1 hyperintensity in S4a/b (arrow) is shown on the pre- and post-contrast T1-weighted image, respectively. Remarkably, the wT1 map reveals subtle, diffuse signal enhancement in S4a/b (arrow) without the use of a contrast agent. The wT1 and wT1_comp_ values of S4a/b (wT1_S4a_ = 814 ms, wT1_comp-S4a_ = 1,000 ms, wT1_S4b_ = 810 ms, wT1_comp-S4b_ = 960 ms) are represented as a thick-edged rhombus (right upper corner wT1 map) in Fig. 4b. wT1, Water T1; wT1_comp_, T2*-compensated wT1

All the above-mentioned 21 liver segments with low wT1 and high wT1_comp_ (thick-edged markers in Fig. 4b, c), corresponding to nine patients, present qualitative fibrosis findings in the T2-weighted or T1-weighted (before or after contrast) images, showing that wT1_comp_ outperforms wT1 in fibrosis tissue signal assessment. For example, Fig. 5 shows a 62-year-old male with idiopathic granulomatous hepatitis. A slight hyperintensity in liver S4a/b is shown on the T1-weighted image, while no abnormalities are detected on the T2-weighted image. Contrast-enhanced T1-weighted fat-saturated axial imaging reveals an area of enhancement within S4a,b. Accordingly, on the proposed wT1 map, a subtle, diffuse signal enhancement is observed in S4a,b. The liver segments of this case are represented as rhombuses in Fig. 4b. After T2*-compensation, S4a,b (thick-edged rhombus in Fig. 4b) present high wT1_comp_, agreeing with the qualitative fibrosis assessment.

**Fig. 6:**
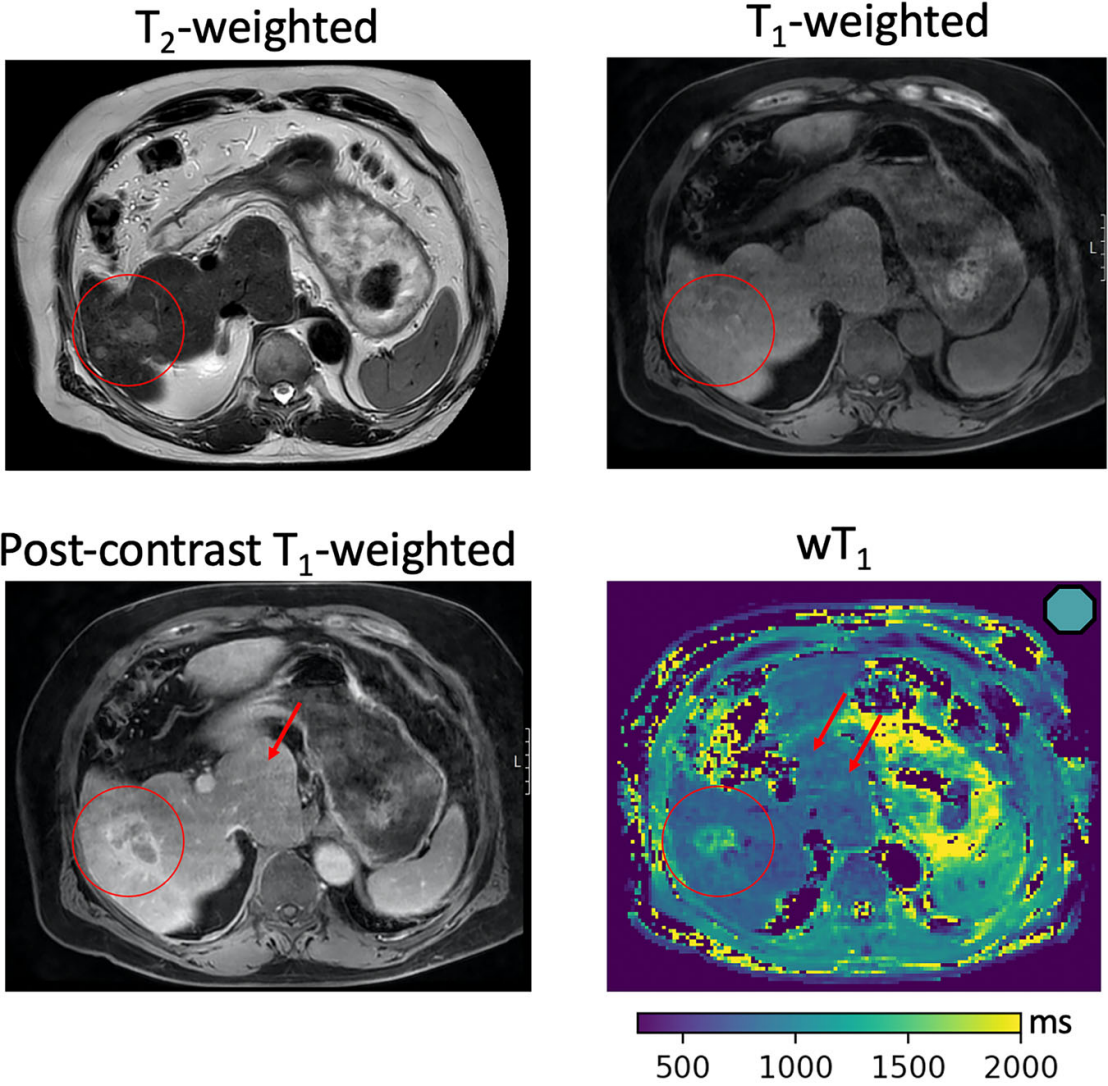
A 73-year-old male with unifocal HCC in S6 (3.0 × 1.8 cm). The HCC (circle) shows hyperintensity on the T2-weighted image, hypointensity on the T1-weighted image and enhancement and washout after gadolinium contrast in the post-contrast T1-weighted image. Reticulations in S1 (arrow) appear post-contrast, with early arterial phase enhancement. On the wT1 map, scars in S1 (arrows) show prolonged T1 relaxation, detectable without contrast agent. The wT1 and wT1_comp_ values of S1 (wT1_S1_ = 893 ms, wT1_comp-S1_ = 988 ms) are represented as a thick-edged octagon (right upper corner wT1 map) in Fig. 4c. HCC, Hepatocellular carcinoma; wT1, Water T1; wT1_comp_, T2*-compensated wT1

**Fig. 7:**
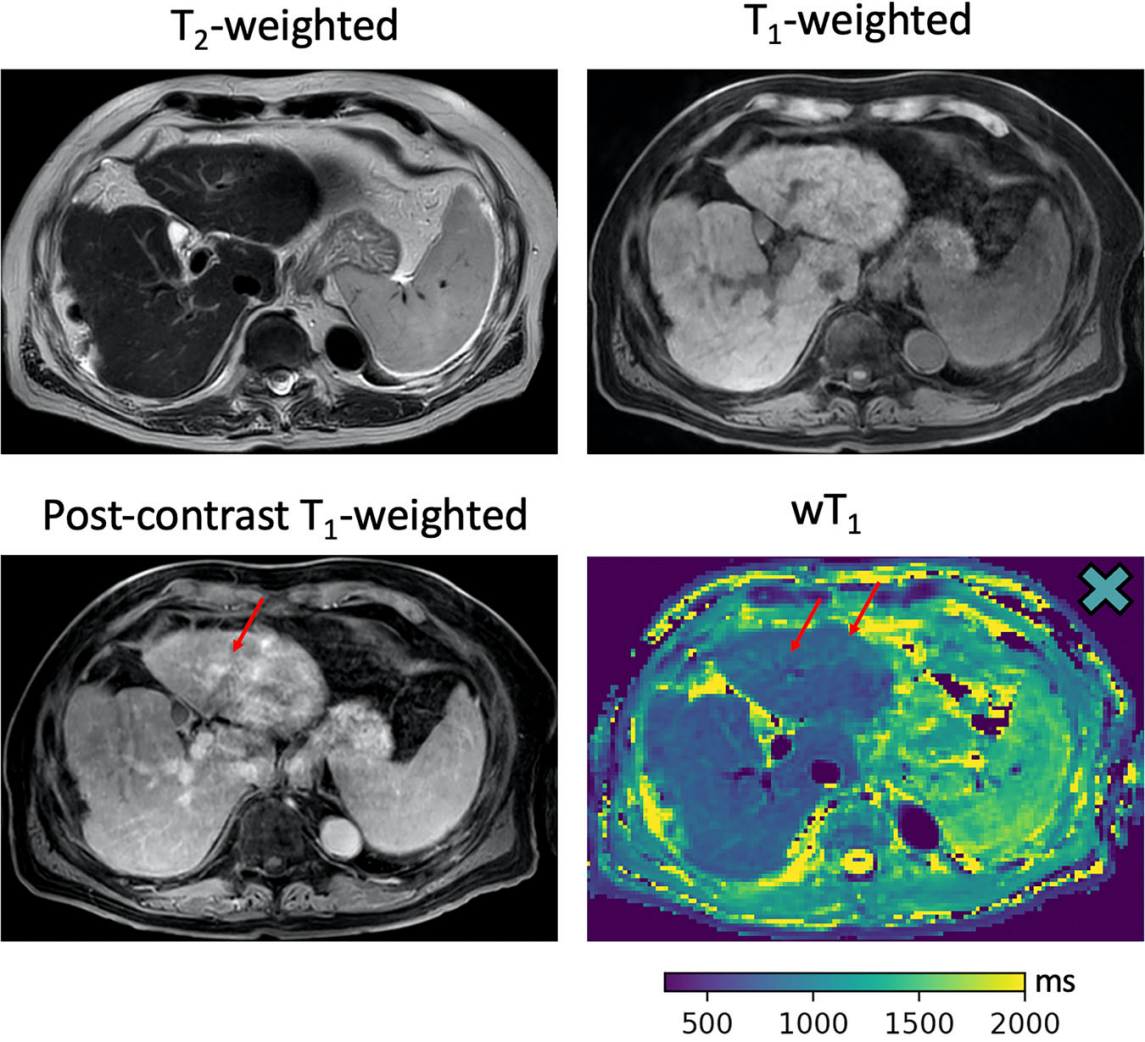
A 77-year-old male with multifocal carcinoma. No hepatic alterations are seen on unenhanced T2- and T1-weighted images. After contrast administration, qualitative fibrosis signs appear in S3 (arrow), while wT1 maps detect scars (arrows) even without contrast application. The wT1 and wT1_comp_ values of S3 (wT1_S3_ = 913 ms, wT1_comp-S3_ = 960 ms) are represented as a thick-edged X (right upper corner wT1 map) in Fig. 4c. wT1, Water T1; wT1_comp_, T2*-compensated wT1

A benefit of the wT1 maps and the proposed T2*-compensation was found for the patients with qualitative fibrosis findings identified in the T1-weighted images, only after the application of the contrast agent. For these cases, the wT1 maps show already differences in the liver segments with and without qualitative fibrosis findings, which become clearer after the T2*-compensation (high wT1_comp_). Figure 6 depicts an unifocal HCC in a 73-year-old male, located in S6, measuring 3.0 × 1.8 cm. The tumor appears hypointense on the unenhanced T1-weighted axial image, demonstrates vivid enhancement after administration of a gadolinium-based contrast agent, and subsequently exhibits washout to hypointensity. No additional abnormalities are detected on the T2-weighted and T1-weighted axial images apart from the HCC. Following contrast administration, reticulations in the liver parenchyma become visible, progressively enhancing over time. While some reticulations in S1 show early enhancement in the arterial phase, most become apparent in later phases. On the wT1 map, scars in S1 exhibit prolonged T1 relaxation and are detectable even without contrast agent administration. Octagons represent the liver segments of this case in Fig. 4c. After T2*-compensation, S1 (thick-edged octagon in Fig. 4c) was the only segment with high wT1_comp,_ corresponding to the qualitative fibrosis findings. Figure 7 displays no clear evidence of hepatic parenchymal alterations on unenhanced T2-weighted and T1-weighted axial images in a 77-year-old male patient with multifocal hepatic carcinoma. After contrast administration, reticulated septa and bridging become visible in S3, while the remaining liver parenchyma shows no signs of fibrosis. Accordingly, on wT1 maps, scars in S3 exhibit prolonged T1 relaxation and are detectable without contrast agent application. In Fig. 4c, the liver segments of this case are displayed as X. Moreover, when applying T2*-compensation, only segment S3 (thick-edged X in Fig. 4c) presented high wT1_comp_, agreeing with the qualitative fibrosis evaluation. These cases underscore the importance of an analysis at the liver segment level when characterizing the liver wT1 values.

## Discussion

The present work evaluates the clinical feasibility of the CIR-LL wT1 mapping method, including all liver segments and the potential of wT1_comp_ for the assessment of qualitative fibrosis findings in benign and HCC patients.

In contrast to recent wT1 mapping techniques, the presented study has shown the wT1 feasibility in benign and HCC patients (Fig. 2), despite the restricted resolution [2.8 × 2.8 × 10.0] mm^3^, and good reproducibility given the high ICC. Moreover, the proposed wT1 maps offer a ${{{{\rm{B}}}}}_{1}^{+}$-robust, broader and faster (1.2-s per slice) liver coverage than previous T1 and wT1 methods by acquiring nine slices in a short breath-hold of 11 s. The inclusion of all liver segments allows for a comprehensive assessment of localized parenchymal changes that might be missed when relying on a limited number of slices. In particular, broad liver coverage can provide valuable insights into fibrosis progression and treatment response, ensuring a more detailed and reliable evaluation of this pathology. Therefore, methods for fibrosis assessment with broad liver coverage, including all liver segments, would be of advantage.

In addition to the water-fat separation, the T2*-compensation is also a fundamental part of the proposed method. The PDFF and T2* ranges (Table 2, Fig. 4) show the broad variation in fat and iron content in the cohort, emphasizing the need to remove these effects. The benign-no-risk group used to estimate the T2*-compensation model includes patients with noninvasive IPMNs and hemangiomas. These patients were selected because they did not present liver lesions and are considered clinically non-malignant. While IPMNs are classified as precancerous, noninvasive forms typically lack the aggressive features of malignancy, making them a suitable benign comparison group in oncologic imaging studies [40]. The similarity of the calculated ${{{\rm{m}}}}$ and ${{{{\rm{mean}}}}}_{{{{{\rm{T}}}}2}^{* }}$ to the results from a healthy group [33] suggests that these parameters might be estimated, in general, from a patient group without liver lesions.

The per-segment analysis is a major contribution of the present work. Previous works about fibrosis T1 [1–3, 5, 9, 20], wT1 [19] and wT1 with T2*-compensation [33] have analyzed their results based on a few liver segments. However, segmental heterogeneity in liver fibrosis has been demonstrated in biliary atresia using ultrasound and shear-wave elastography [41]. Similarly, MRI studies in patients with primary sclerosing cholangitis and viral hepatitis have revealed segment-specific variations, indicating that fibrosis assessment may depend on the liver segments [42]. In accordance, our results show that the liver segments of the same patient can present different values (Fig. 4). Furthermore, it has been demonstrated that texture analysis, including all liver segments, can differentiate low-grade from high-grade fibrosis in 3D CT [43]. Moreover, HCC lesions can induce fibrosis in the perilesional zone, often manifesting as reactive desmoplasia once the tumor has developed [44]. This is supported by the highest mean wT1 values observed in the HCC group in our study (Table 2). Additionally, these findings align with previous research indicating that T1 values are higher around hepatic metastases than around focal benign lesions [45]. Given the liver heterogeneity, the inclusion of all liver segments information would benefit fibrosis analysis.

Based on the liver segmental analysis, the potential of wT1_comp_ for noninvasive contrast-free fibrosis tissue signal assessment has been shown, outperforming wT1. Figures 5–7 illustrate the value of the proposed wT1 mapping method in differentiating hepatic pathologies. While conventional sequences often show limited or nonspecific findings, wT1 maps allow detection of subtle parenchymal alterations and qualitative fibrosis signs, frequently visible even before contrast administration. However, liver segments showing qualitative fibrosis signs can still present low wT1, requiring T2*-compensation to be associated with high wT1_comp_. Liver segments with low wT1, but high wT1_comp_ (Fig. 5–7) underscore the importance of regional liver analysis and support the potential of wT1_comp_ for a comprehensive, segment-specific assessment of liver pathology. The proposed liver segments analysis helps especially to identify a single liver segment that presents qualitative fibrosis signs (octagon and X Fig. 4c, Figs. 6 and 7). A single liver segment with high wT1_comp_ could be wrongly associated with the low wT1_comp_ of the other liver segments if it were not included in the analysis. Moreover, S4 of the liver is particularly vulnerable to fibrosis due to its unique vascular architecture, which might explain its frequent high wT1_comp_ value (Fig. S2). Abnormalities in blood supply can lead to heterogeneous perfusion, predisposing S4 to chronic ischemia and an intensified inflammatory response that promotes fibrotic deposition [46, 47]. By enabling noninvasive contrast-free assessment of both diffuse and focal parenchymal alterations, the proposed method might support early identification of treatment-related liver injury and offer a reliable biomarker for monitoring therapeutic response.

This work presents some limitations. First, the cohort is underpowered and includes only nine subjects in the reference group, while gender analysis has not been addressed. Despite this, this study presents clinical feasibility in benign and malignant cases and a high ICC. A study including a large cohort is required to further evaluate the proposed method for the characterization of focal liver changes. Second, fibrosis was assessed qualitatively in previously identified liver segments using only clinical images and lacks validation against histopathology and MRE. Although the initial identification of liver segments with low wT1, but high wT1_comp_, knowing that fibrosis increases liver T1 values, might influence the reader’s visual assessment, the fibrosis inspection was performed in this order to illustrate cases where wT1_comp_ might provide clinical value. MRE has been used for noninvasive assessment of liver fibrosis, but it is associated with the need for additional hardware [11]. In contrast, the proposed noninvasive CIR-LL wT1 mapping approach incorporates water–fat separation and T2*-compensation, while avoiding biopsy-related risks and enabling rapid multislice liver assessment. While histological validation studies for wT1 and wT1_comp_ are desirable, they remain challenging due to the invasive nature of liver biopsy and its associated risks [48], including bleeding, infection, and tissue seeding, as well as the potential for sampling error and patient discomfort. As an alternative, histological correlation can sometimes be achieved by examining liver tissue taken from the area next to a surgically removed lesion. Third, reference T1 methods like MOLLI were not used in this study. The CIR-LL wT1 method has already been compared to MOLLI and MRE [49]. While the latter proves that wT1 is a more robust marker of liver fibrosis than MOLLI and ECV in a cohort with diffuse liver disease, the present work extends the clinical feasibility analysis of wT1, including oncological cases with focal liver changes. Furthermore, MOLLI was not included in the scan protocol since the acquisition time to capture all liver segments would be long, overstraining the patients. Fourth, the study design is single-center with retrospective patient classification, restricting the generalization of the results, manual ROI placement in the T2*, PDFF and wT1 maps, which can introduce results variability, as well as a ROI analysis using measurements from all liver segments, neglecting the within-subject dependence. The registration of the maps with anatomical clinical images and the use of segmentation software would allow to automate the ROI placement task, reducing variability. Moreover, a multicenter wT1 and wT1_comp_ study with prospective patient classification and statistical analysis that accounts for within-subject variability would strengthen the validity of the results. Fifth, incomplete breath-holds or cardiac motion (last three slices of Fig. 2a) affect the quality of the wT1 maps. Despite the short 11 s breath-hold, 3% of the slices were affected by motion artifacts, including those caused by incomplete breath-holds, which occurred rarely (6 patients representing 14% of all cases). As an alternative, the present CIR-LL wT1 mapping method might be further explored for respiratory-triggered acquisitions due to its fast acquisition time [50].

In conclusion, the proposed CIR-LL wT1 mapping method is feasible for a broad liver coverage of benign and HCC patients, acquiring nine slices in a short-breath-hold of 11 s. The T2*-compensation of wT1, wT1_comp_, and the analysis considering all liver segments show potential for noninvasive contrast-free fibrosis tissue signal assessment over the whole organ.

## Supplementary information

**Additional file 1:** **Fig. S1.** Intraclass correlation coefficient (ICC). ICC for (**a**) T2*, (**b**) PDFF and (**c**) wT1 show good reproducibility for all parameters. **Fig. S2.** Segment-4 results. S4a,b presents frequently high wT1comp. Three and seven liver segments with high wT1comp correspond to S4 for (**a**) benign-risk patients and (**b**) HCC patients, respectively.

## Data Availability

The datasets generated and/or analyzed during the current study are not publicly available due to privacy or ethical restrictions, but are available from the corresponding author on reasonable request.

## Abbreviations

${{{{\rm{B}}}}}_{1}^{+}$: Transmit radiofrequency field
2D: Two-dimensional
3D: Three-dimensional
B0: Main magnetic field
CIR-LL: Continuous inversion recovery Look-Locker
HCC: Hepatocellular carcinoma
ICC: Intraclass correlation coefficient
IPMN: Intraductal papillary mucinous neoplasm
MOLLI: Modified Look-Locker inversion-recovery
MRE: Magnetic resonance elastography
MRI: Magnetic resonance imaging
PDFF: Proton density fat fraction
ROI: Region of interest
SD: Standard deviation
TE: Echo time
wT1: Water T1
wT1_comp_: T2*-compensated wT1
