# Supplementary material for: Clinical feasibility of accelerated whole liver water T1 mapping with T2*-compensation
Source: Eur Radiol Exp. 2026 Mar 4;10:24. doi: 10.1186/s41747-026-00689-z (PMC12961035; doi:10.1186/s41747-026-00689-z)
Supplement: Supplementary file 1 — Additional file 1: Fig. S1. Intraclass correlation coefficient (ICC). ICC for (a) T2*, (b) PDFF and (c) wT1 show good reproducibility for all parameters. Fig. S2. Segment-4 results. S4a,b presents frequently high wT1comp. Three and seven liver segments with high wT1comp correspond to S4 for (a) benign-risk patients and (b) HCC patients, respectively. [file 41747_2026_689_MOESM1_ESM.pdf]

Clinical feasibility of accelerated whole liver water T1 mapping with T2*-compensation

ELECTRONIC SUPPLEMENTARY MATERIAL

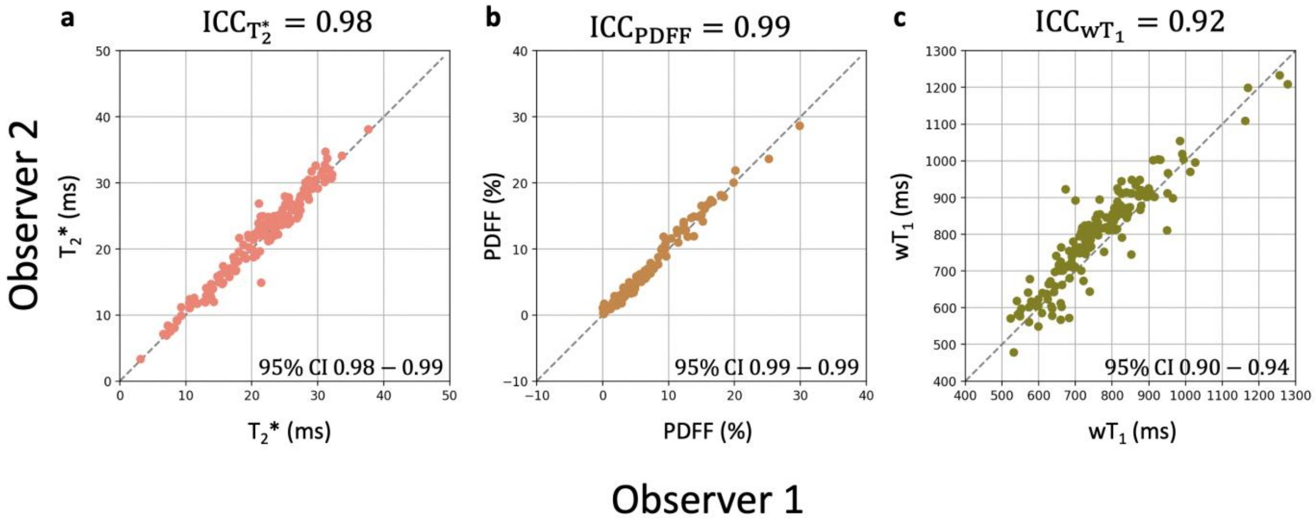

Fig. S1. Intraclass correlation coefficient (ICC). ICC for **(a)** T2*, **(b)** PDFF and **(c)** wT1 show good reproducibility for all parameters. *PDFF P*roton density fat fraction, *wT1* Water T1.

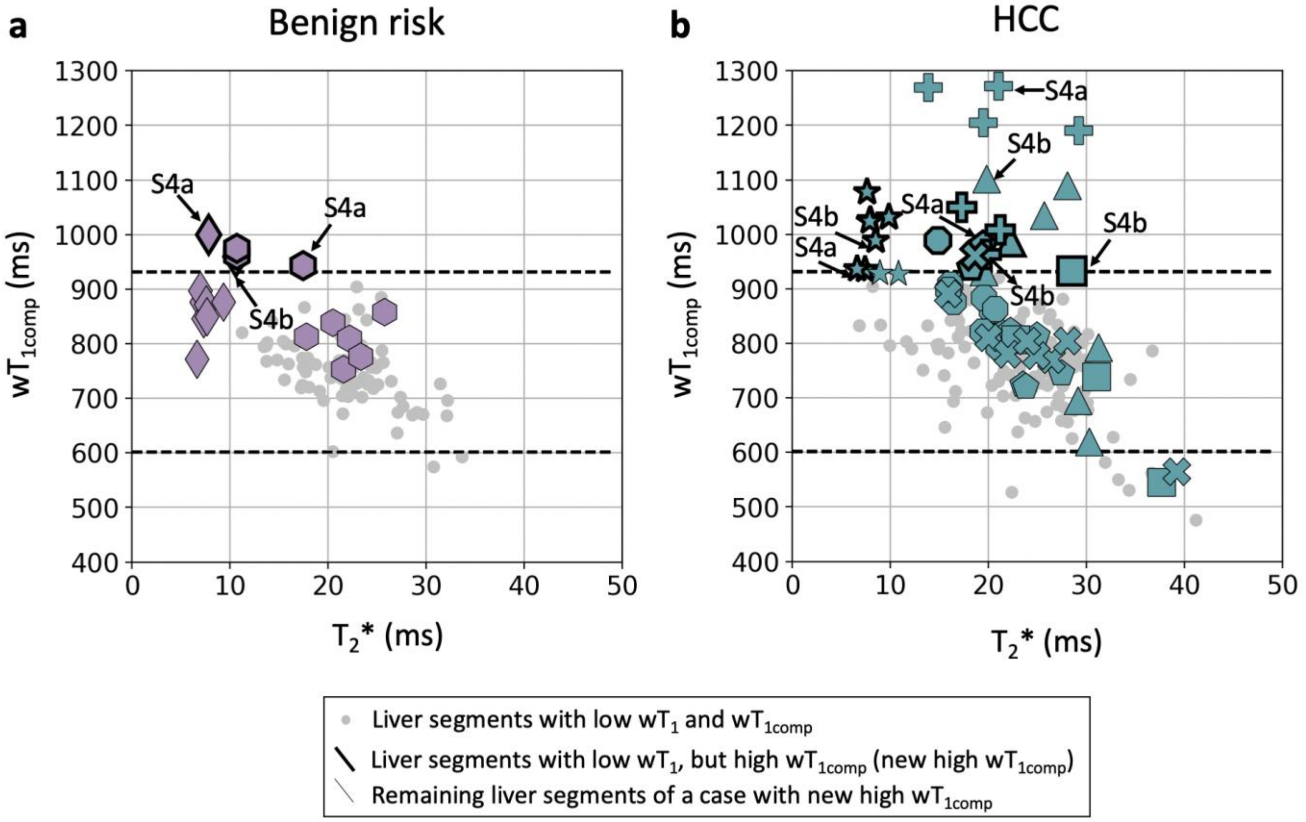

Fig. S2. Segment-4 results. S4a,b presents frequently high $wT1_{comp}$. Three and seven liver segments with high $wT1_{comp}$ correspond to S4 for **(a)** benign-risk patients and **(b)** HCC patients, respectively. *HCC* Hepatocellular carcinoma, *wT1* Water T1, *$wT1_{comp}$* T2*-compensated wT1.
